# Which method is successful in closure of acute oroantral communication? A retrospective study

**DOI:** 10.4317/medoral.26084

**Published:** 2023-12-27

**Authors:** Yusuf Nuri Kaba, Ahmet Emin Demirbas, Cihan Topan, Canay Yılmaz Asan, Beyza Kahraman

**Affiliations:** 1 DDS, Assistant Professor, Department of Oral and Maxillofacial Surgery, Erciyes University Faculty of Dentistry, Melikgazi, Kayseri, Türkiye; 2 DDS, Ph.D., Associate Professor, Department Head, Department of Oral and Maxillofacial Surgery, Erciyes University Faculty of Dentistry, Melikgazi, Kayseri, Türkiye; 3 DDS, Ph.D., Assistant Professor, Department of Oral and Maxillofacial Surgery, Erciyes University Faculty of Dentistry, Melikgazi, Kayseri, Türkiye; 4 Resident, Department of Oral and Maxillofacial Surgery, Erciyes University Faculty of Dentistry, Melikgazi, Kayseri, Türkiye

## Abstract

**Background:**

This study's purpose is to retrospectively evaluate the success of surgical methods used in treating Oroantral Communication (OAC).

**Material and Methods:**

This study was designed as a retrospective cohort study on patients who developed OAC after surgery maxillary posterior region. The records of patients previously treated with OAC were scanned through the hospital registry software. A data set was created by recording patients' age, gender, systemic disease, etiological reasons, and surgical methods. The primary predictor variable was the surgical method used to treat OAC. Other variables were age, gender, systemic disease and etiological reasons. The primary outcome was oroantral fistula development after the first surgical intervention. The patients who were positive in clinical examination and Valsalva test on control days were considered unsuccessful. One-way analysis of variance and Kruskal-Wallis tests were used for quantitative variables in more than two groups. Pearson chi-square test was used to compare categorical data.

**Results:**

This retrospective cohort study was completed with 605 patients who met the study criteria among 95,883 patients who underwent surgery in the maxillary posterior region. The incidence of OAC was 0.63%. The patients consisted of 238 female and 367 male patients. The mean age was 41.06±14.48 years. Buccal flap and Buccal Fat Pad methods were used most frequently in the treatment. While treatment was completed with the first surgical intervention in 592 (97.85%) patients, OAF developed in 13 (2.15%) patients. No statistically significant relation existed between surgical technique and OAF development (*p*>0.005). The success rate of the Buccal Flap method was 98.7%, and the Buccal Fat Pad method was 95.8%.

**Conclusions:**

The results of this study showed that noninvasive methods in openings smaller than 5 mm and surgical treatment methods in openings larger than 5 mm have a high success rate with the limitations of present study.

** Key words:**Oroantral communication, oro-antral fistula, buccal fat pad, buccal flap, plasma rich fibrin.

## Introduction

Oro-antral Communication (OAC) is a relatively rare complication seen during dentoalveolar and maxillofacial surgery. The most common cause of OAC is often the extraction of the maxillary posterior teeth, the anatomically close relationship between the root tips of the premolars and molars and the maxillary antrum, and the thinness of the bone in this region (1). OAC acts as a pathological pathway for bacteria and can cause antrum infection, further hindering the healing process as there is an unnatural communication between the oral cavity and the maxillary sinus. If OACs are not treated, maxillary sinusitis develops in 50% of patients within 48 hours and 90% within two weeks (2). If not diagnosed early and treated promptly, OACs can cause chronic sinusitis, oroantral fistulas, and severe complications. Oroantral fistula (OAF) develops if the OAC remains open and epithelialized.

For this reason, the definitive diagnosis and treatment of OAC is critical to prevent complications and ensure recovery. Immediate closure of OACs, preferably within 24 to 48 hours, is recommended to minimize the risk of maxillary sinusitis and fistula development (3). Many different techniques have been described to close OACs (4). The size of OAC is critical in determining the treatment method. While openings smaller than 2 mm tend to heal spontaneously, openings between 2 and 6 mm can be treated with simple surgical methods such as suturing a gauze pad to stabilize the clot, placing a bleeding stopper, or applying platelet-rich fibrin. Surgical treatment methods such as local flaps (4,5), buccal fat pad (4,6), and grafts (7) are preferred for OACs larger than 6 mm. The most preferred surgical methods are buccal flap and buccal fat pad (8). Surgical treatment of oroantral communication is performed by shifting the mucoperiosteal flap (2,5). However, mucoperiosteal flaps shifted from the buccal region have some disadvantages. Patients experience postoperative pain and swelling. In the long term, the depth of the buccal sulcus decreases, and the compatibility of the denture is impaired (9). Many prospective and retrospective clinical studies in the literature address the success of different methods used to treat OAC (4,10-16). The main limitation of retrospective studies is the small sample size (1,17-19).

This study aims to evaluate the success of surgical methods used in treating OAC in the patient population with a large sample size.

## Material and Methods

- Study Design and Participants

The study was designed as a retrospective cohort study on patients who applied to Erciyes University Faculty of Dentistry, Department of Oral and Maxillofacial Surgery between 2012 and 2023 due to maxillary canine, premolar, and molar tooth extraction. The Erciyes University Clinical Research Ethics Committee (2023/204) approved the study. The study was conducted within the framework of the Human Research Guidelines of Helsinki Declaration. It was conducted according to the Strengthening the Reporting of Observational Studies in Epidemiology (STROBE) statement, Guidelines for Reporting Observational Studies (20). The patients who developed OAC after maxillary posterior surgery had pre-operative panoramic radiographs and undergoing OAC therapy were included in the study. Patients with chronic OAF, congenital syndrome and incomplete records who had previously been operated on because of a tumor or trauma were excluded from the study. The records of patients previously treated with OAC were scanned through the hospital registry software (MedData Tic. ve San. Ltd. Şti., Ankara, Türkiye).

- Study Variables

The primary predictor variable was the surgical method used to treat OAC. The treatment methods were suturing sterile gauze, oxidized cellulosea, plasma-rich fibrin (PRF), buccal flap and buccal fat pad.Other variables age, gender, systemic disease, etiological reasons and size of OAC. Etiological reasons were classified extraction, cycst enucleation, dental implant surgery, osteomyelitis, external sinus lifting, fixation plate removal, alveoplasty, maxillary sinusitis and trauma.

- Study Outcomes

The primary outcome was only OAF development after the first surgical intervention.

- Surgical Procedure

**Figure 1:**
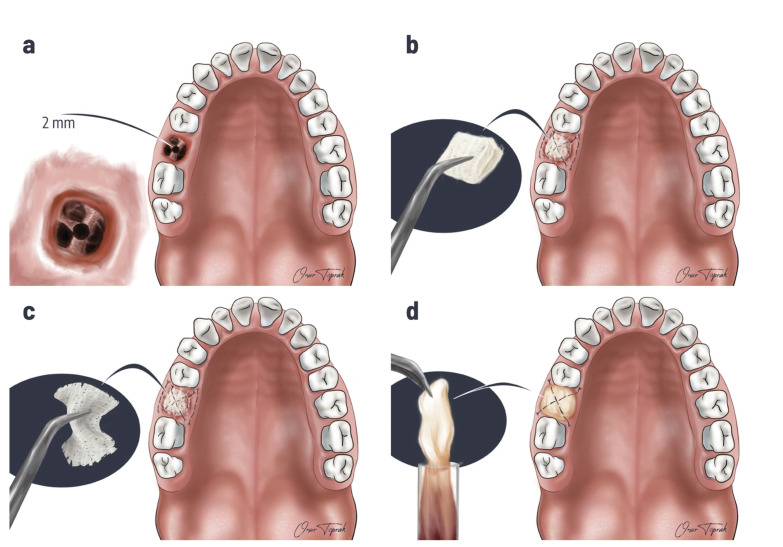
a) OAC between 2 and 5 mm; b) Closure of OAC with Sterile Gauze; c) Closure of OAC with Oxidized Cellulose; d) Closure of OAC with PRF.

**Figure 2:**
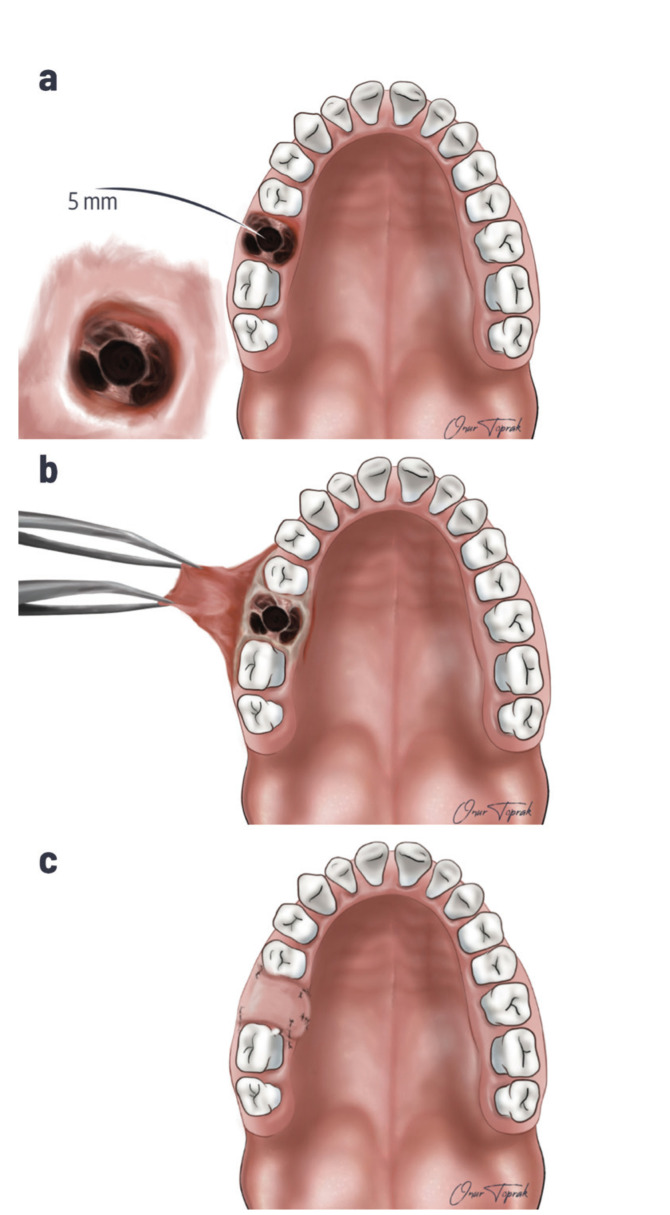
a) OAC larger than 5 mm; b) Preparing Buccal Flap; c) Closure of OAC larger than 5 mm with Buccal Flap.

**Figure 3:**
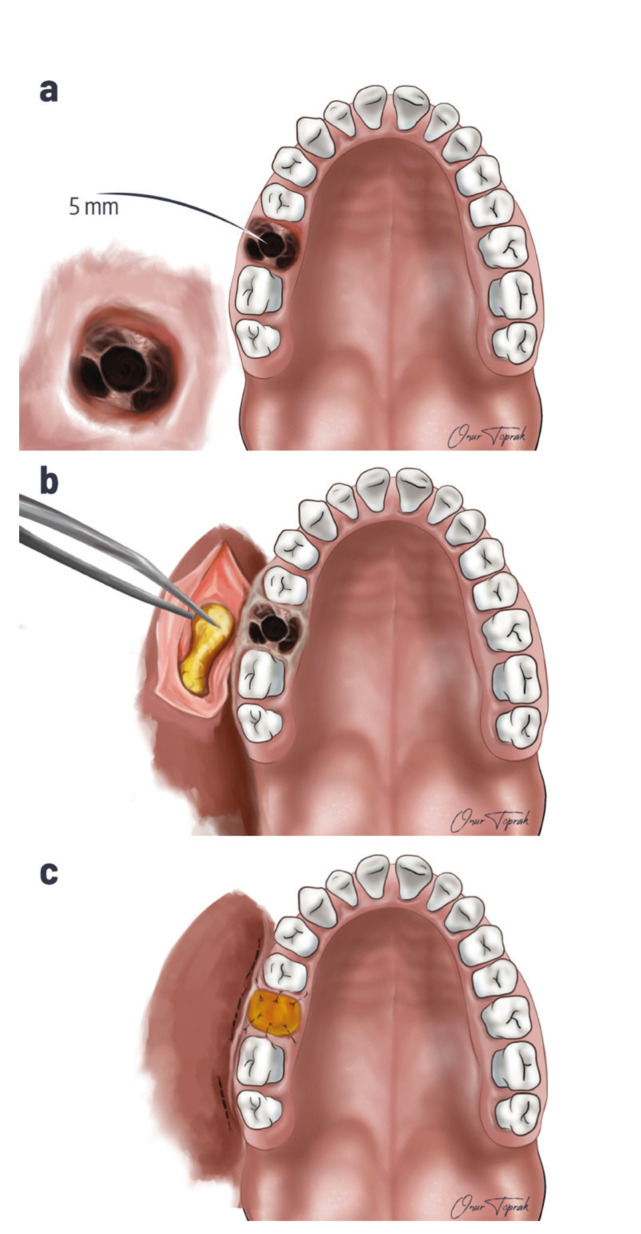
a) OAC larger than 5 mm; b) Preparing Buccal Fat Pad; c) Closure of OAC larger than 5 mm with Buccal Fat Pad.

The diagnosis of OAC was made by clinical examination and the Valsalva maneuver. Cone-beam Computed Tomography (CBCT) images were taken, and the diagnosis was completed in patients who could not be diagnosed by clinical examination. In all patients, OAC was repaired on the same day, immediately after diagnosis. The surgical treatment method was decided according to the size of the opening. The opening size was determined with the help of 2-5 mm diameter curettes or by measuring from CBCT images, if available. In patients with CBCT, the dimensions in the buccolingual direction in the axial section where the opening was widest were measured and recorded in mm. Other patients were classified according to opening size obtained from surgical records.The opening size smaller than 2 mm was allowed to heal spontaneously after the clot was stabilized. If the opening was 2-5 mm (Fig. 1), sterile gauze (Fig. 1), oxidized cellulose (Fig. 1), or PRF (Fig. 1) was fixed to the area with sutures and left to heal. The gauze was removed two days later in patients sutured with sterile gauze. If the opening was more extensive, the patients were treated with Buccal Flap (Fig. 2) and Buccal Fat Pad (Fig. 3).

All patients were prescribed 1 gr amoxicillin + clavulanic acid (twice a day for seven days) and 100 mg flurbiprofen (twice a day for seven days). The patients were advised to avoid movements that would increase the pressure between the mouth and nose. All patients were checked at one week and three weeks after the surgery to ensure that epithelization was completed. Presence of pus drainage, redness, fistula in clinical examination and positive Valsalva test on control days were considered unsuccessful. A second surgery was planned for the treatment of OAF in these patients. A data set was created by recording patients' age, gender, systemic disease, etiological reasons, and surgical methods.

- Statistical Analysis

The standard data distribution was evaluated using histograms, Q-Q plots, and the Shapiro-Wilk test. Descriptive statistics were calculated for each variable. The homogeneity of variance was evaluated using Levene's test. One-way analysis of variance and Kruskal-Wallis tests were used for quantitative variables in more than two groups. The Pearson chi-square test was used to compare categorical data. All data were analyzed using Turcosa Cloud (Turcosa Ltd. Co., Türkiye) statistical software. Differences were considered significant at *p*<0.05.

## Results

The records of 645 patients previously treated with OAC were scanned through the hospital registry software. Forty patients with missing records were excluded from the study. This retrospective cohort study was completed with 605 patients who met the study criteria among 95,883 patients who underwent surgery in the maxillary posterior region between 2012 and 2023. The incidence of OAC was 605/95,883(0.63%). The patients consisted of 238 females and 367 males. The mean age was 41.06±14.48 years. Of the patients, 458 consisted of individuals between the ages of 16-50, and 147 were individuals over 50. While there was no systemic disease in 451 patients, 154 had systemic disease, the most common being hypertension and diabetes. There was hypertension in 39 (6.44%) patients and diabetes mellitus in 33 (5.45%). The demographic data of the patients and their systemic diseases are shown in Table 1. The most common etiological factors in the development of OAC were tooth extraction in 501 (82%, 81%) patients, cyst enucleation in 85 (14.05%), implant in seven (1.16%), osteonecrosis in three (0.5%), external sinus lifting in 2 (0.33%), fixation plate removal in two (0.33%), alveoplasty in two (0.33%), trauma in two (0.33%), and sinusitis in one (0.16%). Data on etiological factors are presented in Table 2.

**Table 1:**
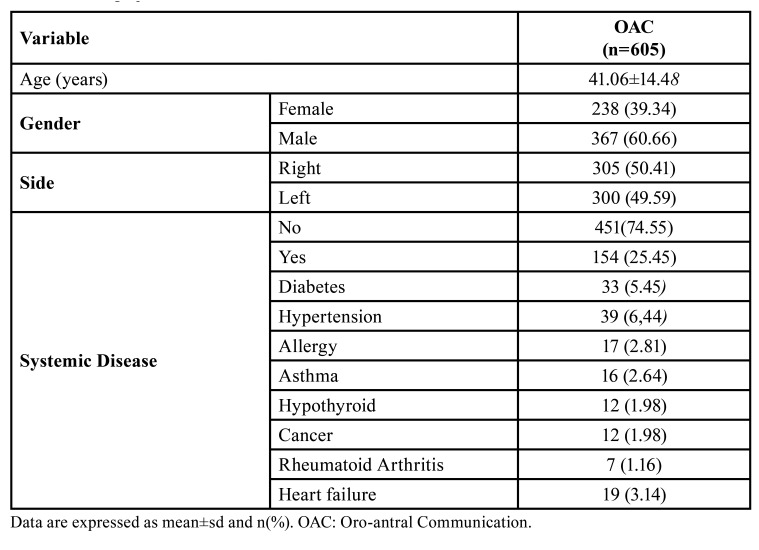
Demographic Data.

**Table 2:**
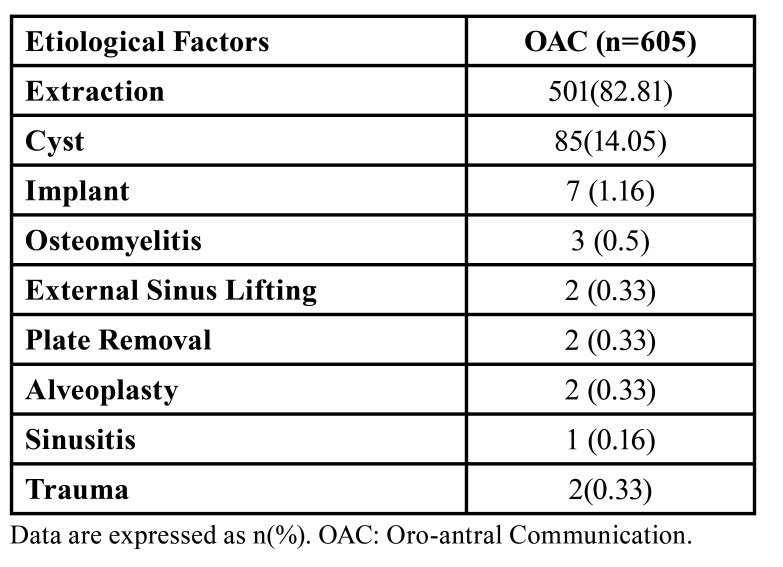
Etiological Factors.

OAC was localized: 251 (41.48%) had the first molar, 137 (22.64%) the second molar, 80 (13.22%) the third molar, 27[4,46] the second premolar, the first premolar in 4(0.66%) and in 2(0.33%) canines in 501 patients who developed OAC due to tooth extraction. OAC was repaired with Buccal Flap in 323 (53.39%) patients, Buccal Fat PAD in 217 (35.88%), Sterile Gauze Suturing in 36 (5.95%), Plasma-Rich Fibrin Suturing in 19 (3.14%), and 10 (1.65%) with oxidized cellulose suturing. OAF development was observed in 13 (2.15%) patients during follow-up, and successful healing was achieved with the first surgical intervention in 592 patients (97.85%). A second surgery was planned for thirteen patients, and OAF treatment was completed. The success rates of the treatment methods are presented in Table 3. There was no statistically significant different relationship between treatment methods in terms of OAF development (*p*=0.230). Among the treatment methods, the most OAF development was seen only in the surgical treatment methods Buccal Fat Pad (4.15%) and Buccal Flap (1.24%). No statistically significant relation was found between treatment methods and the development of OAF (*p*=0.230).

Pre-operative CBCT images were available from only one hundred and one patients treated with Buccal Flap and Buccal Fat Pad. Data on the size of the opening measured on CBCT images are presented in Table 4. The mean age in the patient group with OAF developed was statistically significantly higher than the group without OAF (*p*=0.006). The mean age was 53.46±16.29 years in the group with developed OAF but 40.79±14.33 years in the group without OAF. The size of the oro-antral opening in the Buccal Fat Pad group was statistically significantly higher than in the Buccal Flap group (*p*=0.016). There was no significant relationship between the presence of systemic disease and the development of OAF (*p*>0.05). While nine patients who developed OAF had no systemic disease, one had hypertension+rheumatoid arthritis, one had hypertension+diabetes, and one had asthma. There was no statistically significant relationship between gender and the development of OAF (*p*>0.05).

**Table 3:**
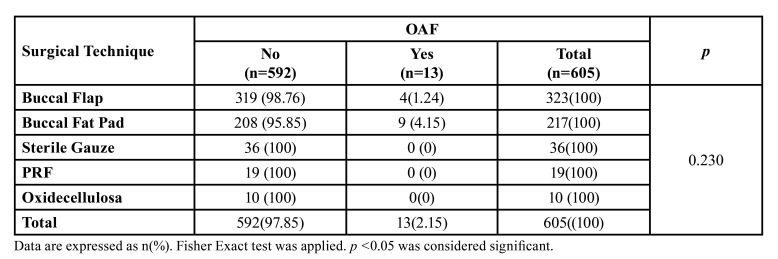
Comparison of Success Rates of Surgical Techniques.

**Table 4:**
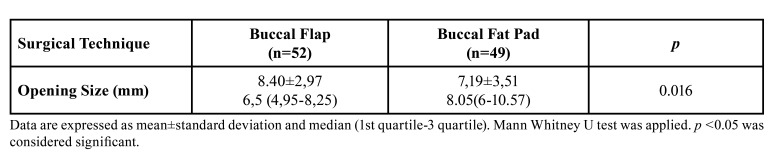
Comparison of Oro-antral Communication Size.

## Discussion

OAC is a rare complication that may occur during surgical interventions in the posterior region of the maxilla. The most common cause of OAC is the extraction of maxillary molars. This study was conducted on 95,883 patients who applied surgery for maxillary posterior region. The incidence of OAC was 0.63% (605/95,883). Demographic features, etiological factors, and success rate of surgical treatment methods in 605 patients who developed OAC were evaluated retrospectively. The most common etiological factor in patients who developed OAC was tooth extraction, followed by cyst enucleation. Buccal flap and Buccal Fat Pad methods were used most frequently in the treatment. While treatment was completed with the first surgical intervention in 592 (97.85%) patients, OAF developed in 13 (2.15%) patients. OAF was observed in four patients with the Buccal Flap groups and nine with the Pedicle Buccal Fat Pad groups. The success rate of the Buccal Flap method was 98.7%, and the Buccal Fat Pad method was 95.8%. In patients who developed OAF, the repair was achieved with a secondary surgery.

The main etiological factor in the development of OAC is tooth extraction (8). In a retrospective study involving 27,984 tooth extractions, OAC was reported in 87 (0.31%) patients. It is most frequently observed in the palatal roots after the first molar tooth extraction. They did not find a statistically significant difference in the incidence of OAC according to gender and age groups (21). Abuabara *et al*. (1), in their retrospective study in 2006, showed that the third molar is the most associated tooth with OAC. They suggested this may be because of the many third molar extractions performed. Studies limited to removing maxillary third molars (surgery) have reported relatively high OAC frequencies of 13%. The risk of OAC was associated with the degree of impaction of the tooth (22). In this study, like previous studies (4,8,18,19), OAC was mainly seen after extracting the first molars, followed by cyst enucleation.

Untreated OAC is a critical problem considering the development of sinusitis, defects that may occur in soft and hard tissues, and the inability to perform implant rehabilitation or pre-implant surgical procedures (23). The process of deciding on an OAC or OAF treatment method depends on many factors, such as the size of the opening, its localization, the time of diagnosis, the presence of infection, a foreign body in the maxillary sinus, and the clinician's experience. It is also affected by the quantity and quality of tissues in the area and the potential placement of future dental implants (24). Parvini *et al*. (25) emphasized in their review that there is a wide range of treatment options and that the most appropriate method should be chosen for the patient. They summarized the factors negatively affecting the success of OAC closure as the size of the OAC, time of diagnosis, inadequate treatment of the pre-operative sinus infection, epithelialization of the fistula tract, and excessive tension on the flap that prevents blood flow for healing.

The general opinion about the dimensions of OAC is that openings smaller than 2 mm heal spontaneously. Tiny perforations in tooth extraction sockets with healthy tissues usually heal if the blood clot is plugged and stabilized in this way. Pressure should be applied to the area with a sterile pack to protect the blood clot. Patients should be informed that they should avoid behavior destabilizing the clot. In the openings between 2-5 mm, it is aimed to provide stabilization with simple surgical interventions due to concerns about clot stabilization. For this purpose, fixing materials such as sterile gauze, plasma-rich fibrin (10), and oxide-cellulose (8) to the area with sutures is frequently preferred. Demetoglu *et al*. (10) reported that the closure of the OAC with a plasma-rich fibrin membrane is less invasive than the buccal flap or Buccal Fat Pad method and preserves the depth of the vestibular sulcus. They showed that the PRF technique is a simple and effective method for treating OACs of 5 mm or less with minimal risk of complications. In the present study, 36 patients were treated by suturing sterile gauze, 19 were treated with plasma-rich fibrin, and 10 were sutured after oxidized cellulose was placed. OAF did not develop in any of these patients.

Many alternative techniques have been developed for treating OAC (26). A study in the literature reports that large OACs are repaired with a collagen membrane (16). One of the most outstanding features of the technique is removing the necessity of covering the membrane and periods in which exposed parts of the membrane epithelialize in their mouths within 14 days. However, this technique has the disadvantage of creating additional costs. Hass *et al*. (26) used mono-cortical bone grafts in OAC equipment. The autogenous graft collected with the trephine bur is placed in the defect area. Although the morbidity of the donor area and the difficulties of the intervention are the disadvantages of this method, it provides an advantage in terms of implant surgery over filling the defect with an autogenous bone graft. Zide *et al*. (27) used OAC treatment by stimulating hydroxyapatite blocks to the defect. The method is easy to apply, does not cause donor site morbidity, and the advantage is that the membrane needs to be entirely operated with a flap.

Despite all these alternative methods, surgeons prefer surgical methods such as Buccal flap, Buccal Fat Pad, and Palatal Flap for openings larger than 5 mm. There are two basic principles to consider when treating OAC and OAF surgically. The first is that the sinus can drain adequately through the ostium without signs of infection. Second, the tissues should be tension-free during closure and consist of a broad-based, well-vascularized soft tissue flap over intact bone. Before closure of OAF, sinus pathology and fistula tract must be excised and degenerated, mucosa and diseased bone must be removed entirely. Many treatment methods have been introduced in the treatment of OACs. Among these, the most common and oldest method is the Buccal flap method (5). Although this method has dependable, effective, and predicTable results, it has disadvantages such as decreased vestibular sulcus depth, postoperative pain, and edema. Sometimes, a second surgery to deepen the vestibular sulcus may be necessary after 6 to 8 months. In some studies, it has been reported that the success rate of Buccal Flap in OAC treatment is below 90% (8,18). The success rate Arx *et al*. (20) reported in 2020 is 95.7%. In the present study, the most preferred treatment method for the treatment of OAC was Buccal Flap. The success rate of the Buccal Flap was 98.76%. OAF developed in four patients who were treated with Buccal Flap, and a second repair was performed with a Buccal Fat Pad in these.

Anatomically, the buccal fat strap, also known as Bichat's fat Pad, is one of the few encapsulated fat masses located between the buccinator muscle on both sides of the face and more superficial muscles such as the masseter, zygomaticus major, and zygomaticus minor (6). The deep buccal and temporal branches of the maxillary artery and the smaller branches of the facial arteries supply the central part of the buccal fat suspension and ensure their successful maintenance in the reconstruction of oral defects (14). Since the first use of the buccal fat wrap flap, its many uses have been for treating OAF due to its success rate and performance. Abuabara *et al*. (1) examined 112 patients with OAF and showed that the success of this technique was 100%. They argued that defects as small as 4 mm were better repaired with simple sutures, but for defects larger than 5 mm, the Buccal Fat Pad was a suiTable method. Poeschl *et al*. (4) used the Buccal Fat Pad to treat OAC in 161 patients and reported a 98% success rate. Gheisari *et al*. (18) reported the success rate of Buccal Fat Pad as 98.3 in spreads greater than 5 mm. The second most preferred method in treating OACs larger than 5 mm was the Buccal Fat Pad in the present study. Buccal Fat Pad was applied in 217 patients, and the success rate was 95.8%. In nine patients who developed OAF, the surgical procedure was repeated, and the OAF was repaired. The mean age of the patients who developed OAF was statistically significantly higher than those who did not (*p*=0.006). No statistically significant relationship was found between gender and the development of OAF. However, the incidence of OAF was higher in men. It may be associated with the higher male distribution in this study. No statistically significant correlation was found between the presence of systemic disease and the development of OAF. Most of the patients who developed OAF did not have systemic disease. Pre-operative CBCT images were available from only one hundred and one patients treated with Buccal Flap and Buccal Fat Pad. The opening size in the Buccal Fat Pad group was statistically larger than the Buccal Flap group (*p*=0.016). The Buccal Fat Pad technique's lower success rate than the buccal flap technique may be related to this.

Due to its retrospective nature, the major limitations of this study are possibility of undiagnosed cases or treated in other clinics after our treatment, lacks the size and localization of the OAC. Prospective, large sample randomized controlled studies are needed to elucidate this relationship fully.

## Conclusions

OAC is a rare complication that occurs after oral surgical procedures. The results of this retrospective study showed that noninvasive methods in openings smaller than 5 mm and surgical treatment methods in openings larger than 5 mm have a high success rate with the limitations of present study. Due to its retrospective nature, the major limitations of this study are possibility of undiagnosed cases or treated in other clinics. Prospective, large sample randomized controlled studies are needed to elucidate this relationship fully.

## References

[1] Abuabara A, Cortez AL V, Passeri LA, De Moraes M, Moreira RWF (2006). Evaluation of different treatments for oroantral/oronasal communications: experience of 112 cases. Int J Oral Maxillofac Surg.

[2] Del Junco R, Rappaport I, Allison GR (1988). Persistent oral antral fistulas. Arch Otolaryngol Neck Surg.

[3] Haanaes HR, Gilhuus-Moe O (1972). Experimental Oro-Paranasal communications. Acta Odontol Scand.

[4] Poeschl PW, Baumann A, Russmueller G, Poeschl E, Klug C, Ewers R (2009). Closure of oroantral communications with Bichat's buccal fat pad. J Oral Maxillofac Surg.

[5] Von Wowern N (1982). Closure of oroantral fistula with buccal flap: Rehrmann versus Moczar. Int J Oral Surg.

[6] Hanazawa Y, Itoh K, Mabashi T, Sato K (1995). Closure of oroantral communications using a pedicled buccal fat pad graft. J oral Maxillofac Surg.

[7] Awang MN (1988). Closure of oroantral fistula. Int J Oral Maxillofac Surg.

[8] Visscher SH, van Roon MRF, Sluiter WJ, van Minnen B, Bos RRM (2011). Retrospective study on the treatment outcome of surgical closure of oroantral communications. J oral Maxillofac Surg.

[9] Güven O (1998). A clinical study on oroantral fistulae. J cranio-maxillofacial Surg.

[10] Demetoglu U, Ocak H, Bilge S (2018). Closure of oroantral communication with plasma-rich fibrin membrane. J Craniofac Surg.

[11] Thoma K, Pajarola GF, Grätz KW, Schmidlin PR (2006). Bioabsorbable root analogue for closure of oroantral communications after tooth extraction: A prospective case-cohort study. Oral Surgery, Oral Med Oral Pathol Oral Radiol Endodontology.

[12] Killey HC, Kay LW (1967). An analysis of 250 cases of oro-antral fistula treated by the buccal flap operation. Oral Surgery, Oral Med Oral Pathol.

[13] Batra H, Jindal G, Kaur S (2010). Evaluation of different treatment modalities for closure of oro-antral communications and formulation of a rational approach. J Maxillofac Oral Surg.

[14] Nezafati S, Vafaii A, Ghojazadeh M (2012). Comparison of pedicled buccal fat pad flap with buccal flap for closure of oro-antral communication. Int J Oral Maxillofac Surg.

[15] Bilginaylar K (2018). The use of platelet-rich fibrin for immediate closure of acute oroantral communications: an alternative approach. J Oral Maxillofac Surg.

[16] Gacic B, Todorovic L, Kokovic V, Danilovic V, Stojcev-Stajcic L, Drazic R (2009). The closure of oroantral communications with resorbable PLGA-coated β-TCP root analogs, hemostatic gauze, or buccal flaps: A prospective study. Oral Surgery, Oral Med Oral Pathol Oral Radiol Endodontology.

[17] Hernando J, Gallego L, Junquera L, Villarreal P (2010). Oroantral communications. A retrospective analysis. Med Oral Patol Oral Cir Bucal.

[18] Gheisari R, Zadeh HH, Tavanafar S (2019). Oro-antral fistula repair with different surgical methods: A retrospective analysis of 147 cases. J Dent.

[19] Von Arx T, Von Arx J, Bornstein MM (2020). Outcome of first-time surgical closures of oroantral communications due to tooth extractions. A Retrosp Anal.

[20] Von Elm E, Altman DG, Egger M, Pocock SJ, Gøtzsche PC, Vandenbroucke JP (2007). The Strengthening the Reporting of Observational Studies in Epidemiology (STROBE) statement: guidelines for reporting observational studies. Lancet.

[21] Punwutikorn J, Waikakul A, Pairuchvej V (1994). Clinically significant oroantral communications-a study of incidence and site. Int J Oral Maxillofac Surg.

[22] Rothamel D, Wahl G, d'Hoedt B, Nentwig GH, Schwarz F, Becker J (2007). Incidence and predictive factors for perforation of the maxillary antrum in operations to remove upper wisdom teeth: prospective multicentre study. Br J oral Maxillofac Surg.

[23] Scattarella A, Ballini A, Grassi FR, Carbonara A, Ciccolella F, Dituri A (2010). Treatment of oroantral fistula with autologous bone graft and application of a non-reabsorbable membrane. Int J Med Sci.

[24] Dym H, Wolf JC (2012). Oroantral communication. Oral Maxillofac Surg Clin.

[25] Parvini P, Obreja K, Begic A, Schwarz F, Becker J, Sader R (2019). Decision-making in closure of oroantral communication and fistula. Int J Implant Dent.

[26] Haas R, Watzak G, Baron M, Tepper G, Mailath G, Watzek G (2003). A preliminary study of monocortical bone grafts for oroantral fistula closure. Oral Surgery, Oral Med Oral Pathol Oral Radiol Endodontology.

[27] Zide MF, Karas ND (1992). Hydroxylapatite block closure of oroantral fistulas: report of cases. J oral Maxillofac Surg.

